# How our advisory board members are using generative AI in teaching

**DOI:** 10.1016/j.patter.2025.101389

**Published:** 2025-10-10

**Authors:** Preeti Patel, Ganesh Mani

## Abstract

Generative AI technologies are creating both new challenges and opportunities for educators around the world. In this People of Data piece, we asked two of the journal’s advisory board members to share how they are using generative AI technologies in teaching and their views about the future of AI in education.

## Main text

### Preeti Patel

The most exciting thing about generative AI in teaching right now is its versatility: it is being explored in countless different ways across classrooms. From design workshops to coding classes, from forensic science to digital marketing, my colleagues are experimenting with generative AI in ways as varied as the subjects themselves, and that variety is where its real value lies.

We are using AI translation tools to stimulate discussions about cultural nuance in language classes. We generate synthetic CCTV images for forensic science case studies to make them more immersive and realistic. In other areas, AI is used to assess student work against rubrics to provide faster, more consistent feedback that students say they prefer. AI is also reshaping classroom activities more broadly. In one exercise, students explore different prompting strategies with ChatGPT and then share insights about its strengths and limitations, reinforcing that AI is not a reasoning machine but a tool with constraints. In design education, co-creation workshops invite students to critically explore ethics and innovation while producing AI-enhanced work, such as films with AI-generated voiceovers, reflecting on the technology’s role in their future industry. We are piloting vibe coding with our students to show what generative AI can do and where it falls short. It lets them build software quickly with prompts while learning to question and verify AI-generated results. In writing classes, students experiment with styling by using AI to see how the same topic can be expressed in different tones, such as persuasive or optimistic. For me, the most interesting part of seeing these practices develop is not their speed or novelty but their potential to nurture critical thinking and metacognition; something that is hopefully reflected in the emerging “study modes” that take a Socratic approach.

These generative AI teaching initiatives, however, do not exist without challenges. One of the conversations we are having is about equity, particularly around financial privilege, whereby some students can afford sophisticated, up-to-date AI tools while others are limited to free versions with restrictions. This creates disparities that we, as educators, need to be aware of; something that, if left unaddressed, could risk widening the digital divide in education.^1^ Another challenge is transparency. Even among researchers, proper citation of AI tools is inconsistent, so it is no surprise that undergraduates struggle to acknowledge their AI use openly. This sometimes creates tension: in one discussion, a business undergraduate said he felt disadvantaged when peers used AI without restriction while a PhD student described how essential it was for planning research and speeding up their thinking. The difference in their experiences shows just how uneven this landscape is. Further, regarding cultural capital, some switched-on students know how to integrate AI seamlessly into their toolkit, often in ways that are hard to detect. Others are unsure or even fearful of using it, and without proper guidance, they can end up in academic misconduct cases through simple misunderstanding. It is clear that access, know-how, and institutional support, or lack of it, are all shaping fair practices. I believe that for generative AI to be meaningfully integrated into education, we need to prioritize ethical clarity and cross-disciplinary collaboration. This is echoed in recent research that highlights both the ethical challenges and the institutional responses beginning to emerge.^2^^,^^3^

On a final point, I think there is a deeper shift we need to acknowledge: AI is no longer just helping students learn; it is becoming part of how they think. As chatbots become constant study companions, the boundary between using AI to learn and using it to live is beginning to blur. That is what makes this moment in education so fascinating and why we need to keep experimenting and learning alongside our students.

### Ganesh Mani

In the business world, we are witnessing an accelerated shift from classical methods of production, sales, and operations planning—hallmarks of Management Science 1.0—to a more dynamic, data-driven paradigm that blends human domain expertise with machine intelligence. By designing projects that mirror this industry evolution, students are encouraged to explore how traditional sales and operations planning can transition into multi-human, multi-machine decision-making environments, where vast volumes of constantly changing data interact with complex algorithms. This shift represents what might be called Management Science 2.0, where machine science builds upon and extends the foundation of management science. For example, in contemporary route optimization,^4^ language models have been used to learn drivers’ delivery behaviors and infer delivery zones while classical algorithms such as the Traveling Salesman Problem are embedded to optimize routing within each zone—demonstrating a seamless integration of behavioral learning and mathematical precision. Similarly, in student capstone team formation, a “vibe optimization” framework^5^ employs large language models to extract project requirements from descriptions and matches them with student preferences and self-reported skills. For the actual matching process, OpenAI’s *o1* model—suitably prompted—expresses the mathematical problem, which is then solved using Python’s PuLP library, allowing for iterative comparison of solution quality. Such examples show how generative AI can effectively blend qualitative insights and preferences with rigorous quantitative frameworks in decision-making.

Generative AI is playing a pivotal role in this transformation. Unlike deterministic decision-making based on fixed logic (e.g., 4 + 3 = 7), generative AI fosters more flexible reasoning under uncertain conditions, even when data or models are incomplete, noisy, or subject to hallucination. Generative systems can also simulate synthetic personas, qualitatively explore “what-if” scenarios, and generate code for very specific applications. In the classroom, generative AI is used to build customized, parameterized case studies that promote “quantamental” reasoning—integrating quantitative analysis (such as net present value or customer lifetime value) with human judgment essential for assessing geopolitical risks or governance challenges. Students are encouraged not just to pursue correct answers but to clearly articulate the collaborative process within their multi-human, multi-machine teams. This approach supports the development of critical thinking, computational and data literacy, and first-principles reasoning—skills integral to successfully practicing Management Science 2.0.

Students are equipped for global careers by being involved in creative, industry-defined projects and collaborative human-machine teaming. Examples include multi-objective portfolio optimization—aligned with the previously mentioned vibe optimization framework—and analyzing customer complaints, whether those complaints originate internally within corporate structures or are submitted publicly to regulators. These experiences help students cultivate the adaptive and analytical mindset required by today’s international business environment.

I have recently created a video for the OpenAI Academy collection that explores how Collaborative AI, developed through decades of practical applications in fields such as health, wealth, and wisdom, enables students to leverage collective intelligence and experiential learning. The video highlights a familiar challenge in consensus building: when many people seek to share ideas, both physical and virtual rooms can leave some voices unheard. Dividing participants into smaller groups streamlines conversation but risks losing valuable cross-group insights. Generative AI addresses this by listening to conversations in various rooms and sharing key discussion points across groups, offering the efficiency of small group interaction while preserving the richness of collective knowledge. This framework, as described by Rosenberg et al.,^6^ has proven to be a powerful teaching tool for illustrating how consensus evolves in large groups. For instance, in one exercise, students allocate a hypothetical investment portfolio across asset classes for two personas—a young, aggressive saver and an older individual approaching retirement—challenging them to balance quantitative analysis with qualitative reasoning.

My teaching also addresses how agent-based frameworks can be layered onto generative AI, as well as the importance of digital public infrastructure and alternative data in crafting holistic and robust solution sets.

## Acknowledgments

G.M. was partially supported by a GAITAR fellowship from the Eberly Center at Carnegie Mellon University.

## Declaration of interests

P.P. and G.M. are members of *Patterns’* advisory board.

## Declaration of generative AI and AI-assisted technologies in the writing process

G.M. acknowledges the use of Microsoft Copilot to assist with language refinement and summarization during the preparation of this manuscript. The author is solely responsible for the content and interpretation of the work. Portions of the article were refined by P.P. using AI assistance (ChatGPT-4) to maintain conciseness within the required word limit.
